# Liver disease during and after hematopoietic stem cell transplantation in adults: a single-center Egyptian experience

**DOI:** 10.1186/s43046-020-0020-1

**Published:** 2020-02-22

**Authors:** Haitham Abdelbary, Rasha Magdy, Mohammed Moussa, Inas Abdelmoaty

**Affiliations:** Department of Clinical Hematology and Bone Marrow Transplantation, Ain Shams University, 56 Ramsis street, Abbasia, Cairo, Egypt

**Keywords:** Hematopoietic stem cell transplantation, Hepatotoxicity, Graft versus host disease, Sinusoidal obstruction syndrome

## Abstract

**Background:**

Hepatic complications are a well-known cause of both early and late mortality and morbidity in hematopoietic stem cell transplant (HSCT) recipients. Early diagnosis and management of hepatic complications is important in order to commence appropriate therapy. Conditioning regimens, acute and chronic graft versus host disease, sinusoidal obstruction syndrome, and infections among others represent major hepatic complications for the transplant recipient. We assessed liver function tests, viral markers, polymerase chain reaction, abdominal ultrasound, portal, and hepatic venous duplex in 88 patients underwent autologous and 102 patients underwent allogeneic transplant as well as liver biopsy in selected patients in this retrospective study and evaluated early and late hepatic complications and their impact on transplant outcome.

**Results:**

The major cause of hepatic injury in allogeneic patients is the conditioning regimen (38.8%) followed by acute GVHD (14.7%), after day +100 chronic hepatic GVHD is the primary cause of liver injury which occurred in about 40% of allogeneic patients. In autologous patients, the first cause of hepatotoxicity is also conditioning regimen involving 27.9% of patients followed by flare of viral hepatitis in 7.9% and sepsis in 6.3% of cases. The prevalence of HCV, HBV, and CMV is 19%, 16%, and 8%, respectively.

**Conclusion:**

In our study, conditioning regimens, acute and chronic hepatic GVHD are frequent causes of hepatic injury following allogeneic HSCT while conditioning regimens, flare of viral hepatitis, and sepsis represent the most common causes of hepatic injury following autologous HSCT.

## Background

Hematopoietic stem cell transplantation (HCT) is a curative modality for a wide variety of hematologic disorders. Over the last decades, the safety of HSCT has been improved and the growing use of haploidentical and cord blood transplantation has made it possible to find suitable allogeneic donors and to expand the HCT to older patients [1].

Early survival after HSCT is dependent on the occurrence of hepatic, cardiac, and pulmonary complications and whether serious infections could be avoided [2].

Several comorbidity indices have been designed to assess hepatic dysfunction before transplantation, the most frequent is the hematopoietic cell transplantation comorbidity index (HCT-CI) [3].

Clinical data together with radiologic and laboratory tests are used in current practice to evaluate hepatic dysfunction before transplantation. Among these, AST, ALT, and bilirubin are the most commonly considered in clinical practice and in pre-transplantation predictive models [4, 5].

In the early post-transplant period, infections, drug toxicity, sinusoidal obstruction syndrome (SOS), and acute graft-versus-host disease (aGVHD) are the most frequent causes of hepatic dysfunction while chronic viral hepatitis, chronic GVHD (cGVHD), and iron overload states are the most frequent causes of late liver abnormalities [6].

The aim of this retrospective study was to identify the early and late hepatic complications of hematopoietic stem cell transplantation, the clinical significance of each liver function test abnormality before transplantation, their impact on transplantation outcomes.

## Study design

### Patients and control

The study was a retrospective study that included 190 adult patients who had autologous or allogeneic HSCT between October 2004 and October 2016 at a hematopoietic stem cell transplantation unit at a tertiary hematologic center, after taking informed consent.

Participants were grouped into the following:

- Group I: this group included 88 adult patients who underwent autologous HSCT.
- Group II: this group included 102 adult patients underwent allogeneic HSCT.

Informed consent was obtained from all participants. The study was conducted in accordance with the stipulations of the local ethical and scientific committees of our tertiary care center and the procedures respected the ethical standards in the Helsinki Declaration of 1964.

## Methods

All patients were subjected to full history taking, clinical examination, laboratory or radiological (pre-, during, or post-) transplant workup.

### Pre-transplant workup

#### Laboratory

1. Disease-specific labs:
  Bone marrow aspiration, flow cytometry, and cytogenetic study to detect minimal residual disease before transplant.
  Disease-specific labs, e.g., multiple myeloma patient had serum protein electrophoresis and immunoglobulin quantitation.
2. ABO blood type and (HLA typing needed for allogeneic patients only).
3. Infectious disease screening for hepatitis (A, B, C) testing, HIV (human immunodeficiency virus), HSV (herpes simplex virus), CMV (cytomegalovirus), and EBV (Epstein-Barr virus) by both serology and PCR (polymerase chain reaction), toxoplasmosis.
4. Laboratory tests to check organ function:- CBC, kidney, and liver function (AST, ALT, alkaline phosphatase, γGT, albumin, total, and direct bilirubin), coagulation profile (PT, PTT, INR), serum ferritin level, and pregnancy test for females in child-bearing period.
5. Liver biopsy in selected cases who had pre-transplant liver cirrhosis or fibrosis to assess its grade.

#### Radiological

Chest X-ray and pulmonary function tests, echocardiography and electrocardiogram, pelvi-abdominal ultrasound, computed tomography scan (CT Scan) to detect any residual disease before transplantation, MRI (magnetic resonance imaging) on selected part of body according to patient disease, e.g., MRI brain if there was any suspect of CNS infiltration, positron emission tomography (PET) scan to detect any residual disease before transplant mainly done in lymphoma.

### During and post-transplant workup

#### Laboratory

CBC, kidney function, and liver function, (PT, PTT, INR) is done weekly, CMV PCR is done weekly, cyclosporine level is done weekly, hepatitis B, C PCR, and EBV PCR in selected cases, serum ferritin level, bone marrow aspiration and (chimerism needed for allogeneic patients only) in D28, D90, D180, D270, D360, and liver biopsy for histologic confirmation of hepatic GVHD in selected cases who had allogeneic HSCT.

#### Radiological

In selected cases, for example, pelvi-abdominal ultrasound, hepatic veins, and portal vein duplex in case of suspecting veno-occlusive disease.

### Protocols

Conditioning regimen, GVHD prophylaxis, and supportive care: the conditioning regimens used for allogeneic HSCT were the following:

1. Busulfan 1 mg/kg/6 h PO (day 7 till day 4) in combination with fludarabine 30 mg/m^2^ (day 6 to 3)**.**
2. Busulfan 1 mg/kg/6 h PO (day 7 till day 4) in combination with cyclophosphamide 60 mg/kg day 3 and day − 2 with mesna prophylaxis.
3. TBI one fraction daily (2.5 Gy) for 4 consecutive days in combination with cyclophosphamide 60 mg/kg (day − 3 and day − 2) with mesna prophylaxis.

### Autologous HSCT

The conditioning regimens used for autologous HSCT were the following:

1. Melphalan 200 mg/m^2^ IVI over 30 min on day − 2 was the standard conditioning regimen used in myeloma patients in our center. Patients with impaired renal functions (Cr.clearance < 30 ml/min) received 140 mg/m^2^ of melphalan.
2. Cyclophosphamide 60 mg/kg (day − 3 and day − 2 with mesna as prophylaxis) in combination with etoposide (15 mg/m^2)^ day − 2 and day − 1 and carboplatin 400 mg/m^2^ (day − 3, − 2).
3. Melphalan 200 mg/m^2^ (day − 2) and Etoposide 1600mg/m^2^ (day − 2).

### GVHD prophylaxis

In allogeneic patients, GVHD prophylaxis included cyclosporine (CSA) plus methotrexate (MTX). CSA was started on day − 1 at a dose adjusted to trough blood levels (between 200 and 300 ng/mL). MTX was administered on days + 1, + 3, + 6, and + 11 (10 mg/m^2)^ followed by folinic acid rescue. In vivo T cell depletion with antithymocyte globulin (ATG) was used in patients with aplastic anemia mainly.

### Supportive care

Acyclovir, fluconazole/voriconazole, and quinolones (ciprofloxacin or levofloxacin) were administered from day − 1 until neutrophil recovery as infectious prophylaxis. CMV screening using PCR for guiding pre-emptive therapy. Galactomannan in blood samples was performed in suspected cases of neutropenic patients who are not responsive to neutropenic fever protocol ursodeoxycholic acid used as prophylactic agent to prevent liver injury.

### Hepatic dysfunction

The medical records of all patients were reviewed and details of serial liver function tests during the pre-transplant and post-transplant period were evaluated. These data were collected and analyzed for specific time periods after transplantation: − 1 to 30 days, 31 to 100 days, and 101 to regular follow-up as long as the patient is alive.

For most patients, liver function tests were carried out twice weekly during the first 30 days, were done daily in critically ill patients. Twice weekly during the next 30 to 60 days, once a week after 60 days to 180 days, and every month after 180 days to 1 year. Note was made of relevant clinical findings and subsequent investigations. In case of liver test abnormalities, patients underwent imaging studies (ultrasonography or computed tomography scan) and microbiological studies (CMV antigenemia/PCR, blood cultures, and other viral PCR studies.

The etiology of abnormal liver function and any therapeutic action taken were recorded and interoperated and classified by using diagnostic criteria of Forbes et al. [7].

1. Hepatic GVHD: the presence of clinical and histological evidence of GVHD in conjunction with the development of abnormal serum liver biochemistry. Acute and chronic GVHD were divided by a temporal cut-off of 100 days after BMT.
2. Drug hepatotoxicity:
  - Drug hepatotoxicity from conditioning therapy: transient development of abnormal liver function during the first 2 weeks after BMT in the absence of other identifiable causes.
  - Cyclosporin hepatotoxicity: clinical features of cyclosporin toxicity (for example, fluid retention, hypertension, renal impairment, and tremor) and elevated serum cyclosporin level (whole blood cyclosporin level ˃ 400 mg/l) in conjunction with the transient development of abnormal liver function.
  - Hepatotoxicity from other drugs: transient abnormal liver function temporally related to the introduction of a drug known to cause hepatotoxicity.
3. Viral hepatitis: development of abnormal serum liver enzymes in conjunction with serological evidence of active viral infection.
4. Sepsis: development of/or deterioration of liver function during an episode of bacterial or fungal sepsis.
5. Veno-occlusive disease: clinical criteria of veno-occlusive disease developed by the Baltimore group were used. The Baltimore criteria include jaundice (bilirubin > 2.0 mg/dl) and two of the following: hepatomegaly (usually painful), ascites, or more than 5% weight gain.
6. Disease recurrence: development of abnormal liver function at a time of clinical evidence of disease recurrence.

### Statistical analysis

The primary endpoint of the study was to determine the impact of liver function abnormalities on the outcome of autologous and allogeneic HSCT. Secondary endpoints were to classify and describe hepatic dysfunction after transplant, and to determine its frequency and risk factors. The incidences of hyperbilirubinemia, acute GVHD, and chronic GVHD were calculated using cumulative incidence estimates. All statistical analyses were performed using SPSS, version 17.0 (SPSS, Chicago, IL, USA).

## Results

One hundred ninety patients were included in this study (88 patients underwent autologous and 102 underwent allogeneic transplant), the type of disorders and the conditioning regimens used were illustrated in Table 1.

**Table 1:** Clinical and laboratory data of patients who had HSCT in the BMT unit

Variable		
Sex	Male/female	121/69
Type of transplantation	Autologous	88 (46%)
	Allogeneic	102 (54%)
Type of illness	Multiple myeloma	47(24.7%)
	AML	44(23.1%)
	Lymphoma	41(21.5%)
	ALL	30(15.7%)
	Severe aplastic anemia	16 (8.4%)
	Chronic myeloid leukemia	7 (3.7%)
	Myelodysplastic syndrome	5 (2.6%)
Virology pre-transplant	HBV	31(16.3%)
	HCV	36 (19%)
	CMV	15 (8%)
Mobilization protocol	VAD	23(22.3%)
	DHAP	38(36.9%)
	Velcade/dexamethasone	31(30.1%)
	Endoxan/G-CSF	38(36.9%)
Conditioning regimen for autologous transplant	Melphalan	45(51.1%)
	Carboplatin/VP16/CY	19(21.5%)
	Melphalan/VP16	16(14.7%)
Conditioning regimen for allogeneic transplant	BU +FLU	51 (50%)
	BU +CY	16(15.6%)
	TBI +CY	13(12.7%)
	CY+FLU+ATG	10 (9.8%)

**Fig. 1:**
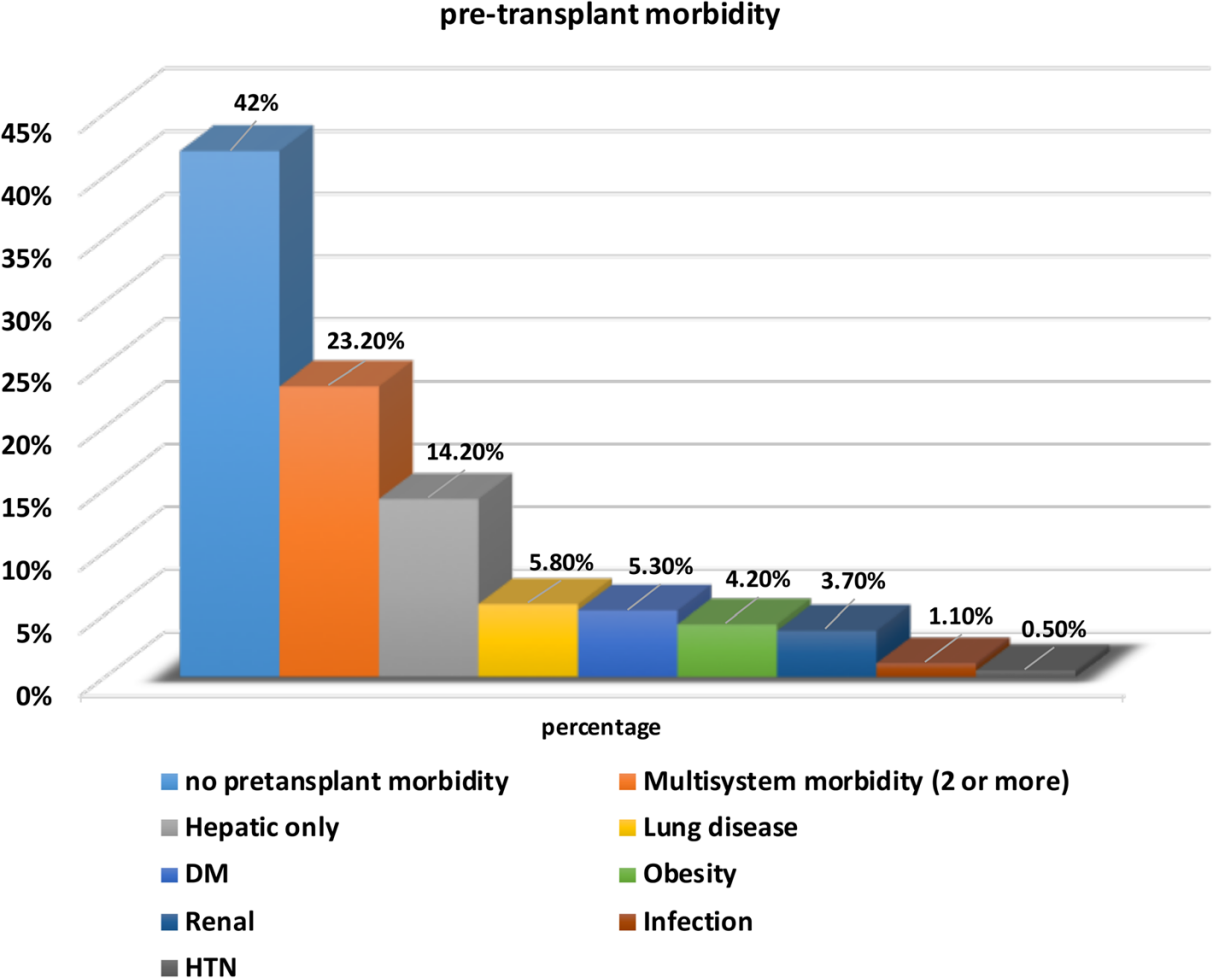
Morbidity pre-transplant

**Fig. 2:**
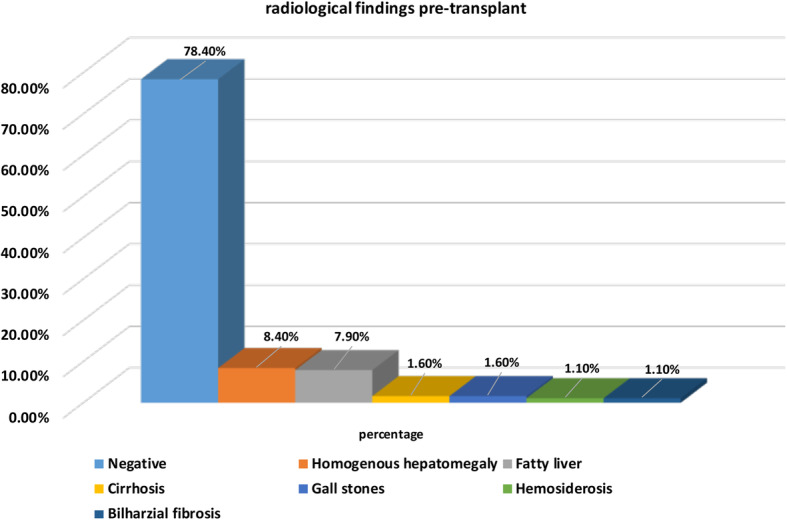
Radiological findings in patients before BMT

Almost half of the patients had no pre-transplant morbidity followed by multisystem involvement in 23.2% and hepatic affection in 14.2% (Fig. 1). Using (abdominal US, CT, or MRI abdomen or PET scan), the main radiological finding was non-specific hepatomegaly followed by fatty livers but most of the patients had no liver involvement (Fig. 2).

Pre-transplant liver injury detected laboratory investigations had a highly statistically significant effect on the liver injury during transplantation (from the onset of conditioning regimen to the time of engraftment) (*P* value 0.000) (Table 2), while pre-transplant liver injury detected laboratory investigations had a statistically insignificant effect on liver post-transplantation (within a 1-year follow-up period) (*P* value 0.138).

**Table 2:** Correlation between abnormal laboratory liver functions pre- and during HSCT

	Positive liver injury during transplant		Negative liver injury during transplant		*χ*^*2*^	*P*
	*N*	%	*N*	%		
Liver affection pre-transplant (positive)	37	19.4	15	7.8	14.128	.000
Liver affection pre-transplant (negative)	56	29.4	82	43.1		
Total	93	49	97	50		

In allogeneic patients during the first 100 days, hepatotoxicity from conditioning regimens occurred in 38.2% and acute GVHD in 14.7% while chronic GVHD occurred in 35.3% (Table 3). In autologous patients, hepatotoxicity from conditioning regimens occurred in 27.9% and flare of viral hepatitis in 7.9% (Table 4).

**Table 3:** Etiology of liver injury and abnormal liver function test

Variable		
Abnormal pre-transplant liver function	Grade I Grade II Grade III Grade IV	14 (7.4%) 7 (3.7%) ------- ------
Abnormal liver function during transplant	Grade I Grade II Grade III Grade IV	53 (27.9%) 15 (7.9%) 12 (6.3%) 12 (6.3%)
Abnormal liver function post-transplant	Grade I Grade II Grade III Grade IV	44(23.2%) 15 (7.9%) 5 (2.6%) 10 (5.3%)
Classification of liver injury in allogeneic patients from D0 to Day + 100	Drugs/conditioning regimen Acute GVHD Flare of viral hepatitis Sepsis SOS CMV hepatitis	39(38.2%) 15(14.7%) 2 (2%) 2 (2%) 2 (2%) 2 (2%)
Classification of liver injury in allogeneic patients after day + 100	Chronic GVHD Double pathology Drugs/conditioning regimen Flare of viral hepatitis Iron overload Sepsis	36(35.3%) 12(11.8%) 4 (3.9%) 2 (1%) 3 (2.9%) 2 (2%)
Classification of liver injury in autologous patients in the early post-transplant period	Drugs/conditioning regimen Flare of viral hepatitis Sepsis	53(27.9%) 15 (7.9%) 12 (6.3%)
Classification of liver injury in autologous patients in the late post-transplant period	Drugs/conditioning regimen Flare of viral hepatitis Sepsis	10(11.4%) 1 (1.1%) 1 (1.1%)

**Table 4:** GVHD sites, grades, and liver biopsy

Variable		
Classification of acute GVHD	Negative Positive GIT alone Positive hepatic alone Positive mucocutaneous alone Combined 2 organs or more	63 (67%) 10 (10.6%) 6 (6.4%) 2 (2.1%) 13 (13.9%)
Grades of acute GVHD	Grade I Grade II Grade III Grade IV	2 (6.4%) 6 (19.3%) 10 (32.2%) 13 (41.9%)
Classification of chronic GVHD	Negative Positive GIT alone Positive hepatic alone Positive mucocutaneous alone Positive pulmonary alone Combined hepatic with another system	25 (30.9%) 3 (3.7%) 13 (16%) 5 (6.2%) 2 (2.5%) 33 (41%)
Grades of chronic GVHD	Grade I Grade II Grade III Grade IV	4 (7.1%) 13 (23.2%) 18 (32.1%) 21 (37.5%)
Liver biopsy	Not done Positive for GVHD Positive for iron overload Combined GVHD and iron overload	65 (76.5%) 14(16.5%) 2 (2.2%) 4 (4.7%)

Acute GVHD occurred in 33% of patients with grade III and IV occurred in 32.2% and 41.9%, respectively, while chronic GVHD occurred in nearly 70% of patients with grades II, III, and IV GVHD occurred in 23.2%, 32.1%, and 37.5%, respectively (Table 5).

**Table 5:** Busulfan-induced hepatotoxicity

	Positive injury of the liver during transplant		Negative injury of the liver during transplant		*χ*^*2*^	*P*
	*N*	%	*N*	%		
Bu-based conditioning	26	25.5	23	22.5	8.777	0.003
Non-Bu conditioning	13	12.7	40	39.3		
Total	39	38.2	63	61.8		

Busulfan-based conditioning regimens were the most common regimen associated with hepatotoxicity compared to non-busulfan based regimens with a highly statistically significant difference (*P* value 0.003) (Table 6).

**Table 6:** Specific liver injury post-allogeneic transplantation

	Specific liver injury post-allogeneic transplantation				Total		Chi-square	
	Positive liver affection		Negative liver affection					
	*N*	*%*	*N*	*%*	*N*	*%*	*χ*^*2*^	*P*
Chronic GVHD (positive)	48	59.2	8	9.8	81	100	26.414	0.000
Chronic GVHD (negative)	7	8.7	18	22.3				
Iron overload (positive)	11	12.7	4	4.7	86	100	0.864	0.352
Iron overload (negative)	43	50	28	32.6				

Chronic hepatic GVHD is the main cause of hepatotoxicity post-transplant with a highly statistically significant difference (*P* value 0.00), while hepatic hemosiderosis is statistically insignificant with hepatic injury post-transplant (*P* value 0.352) [7].

Septicemia had a statistically insignificant effect on the liver during transplant in both autologous and allogeneic patients (*P* value 0.881), but it has a highly statistically significant difference between autologous and allogeneic patients post-transplant, with sepsis being higher in allogeneic patients.

## Discussion

Hepatic dysfunction is a common problem in HSCT recipients. Diagnosis can be accomplished by an interpretation of the clinical setting in which liver dysfunction occurs in order to institute appropriate therapy.

In the current study, the prevalence of pre-transplant liver function abnormalities in both allogeneic and autologous patients before conditioning is 11.1% that increased to 48.8% after conditioning regimens of which 12.6% had grade III–IV hepatic dysfunction. The prevalence is similar to a previous report by El-Sayed et al. from Egypt who found that 10% of patients had mild hepatic dysfunction pre-transplant that increased after conditioning to 47% [8]. Moreover, the previous report by Wang et al. showed a higher percentage of liver function abnormalities pre-transplant (19.8%) that increased to 81% during conditioning regimen [9].

Various observations suggest that patients with hepatic dysfunction before transplantation may be at risk of hepatic disease post-transplant. Others reported that hepatic disease at the time of transplantation did not appear to increase the risk [2].

When we correlated between pre-transplant abnormal transaminase levels and the rise in liver transaminases during transplantation (from day 0 to day + 30), we found a highly statistically significant difference (*P* value 0.000). Conversely, we found that the incidence of long-term hepatic complications was independent of abnormal transaminases pre-transplant.

In our study, the major cause of hepatic injury in allogeneic patients is the conditioning regimen (38.8%) followed by acute GVHD (14.7%) of which 32.2% had grade III and 41.9% had grade IV, respectively. Then similarly, 2% of patients had a flare of viral hepatitis, sepsis, SOS, or CMV hepatitis. These figures are dramatically changed after day + 100 as chronic hepatic GVHD is the primary cause of liver injury which occurred in about 40% of allogeneic patients of which (7.1% had grade I, 23.2% had grade II, 32% had grade III, and 37.5% had grade IV) followed by significant decrease of drug-induced hepatotoxicity (3.9%), iron overload in 2.9%. In autologous patients, the first cause of hepatotoxicity is also conditioning regimen involving 27.9% of patients followed by a flare of viral hepatitis in 7.9% and sepsis in 6.3% of cases. Long-term, drug-induced hepatotoxicity involved 11.4% of patients and is the major cause of hepatotoxicity.

In a similar study, Barba et al. found that acute hepatic GVHD was found in 17% of patients who underwent allo-HSCT while chronic hepatic GVHD was found in 50% of their cohort [3]. Another multicenter retrospective study by Battipaglia et al. that included 146 adults with AML and receiving allo-HSCT, the main cause of liver function abnormalities among patients were acute GVHD (31%), chronic GVHD at 2 years was 25% [10].

Regarding the type of conditioning regimen and hepatotoxicity, we found that busulfan-based conditioning regimens had a statistically significant hepatotoxic effect compared to non-busulfan conditioning regimens.

The population of Egypt has a heavy burden of liver disease. The overall prevalence of positive antibody testing to hepatitis C in the general population is around 15–20% [11]. The prevalence of HCV in our patients is 19% while the prevalence of HBV was 16% and CMV is 8%. Similarly, the previous report by El-Sayed et al. from Egypt with similar environmental factors revealed that the prevalence of pre-transplant HBV and HCV in their patients was 27% and 31%, respectively [8]. The flare of viral hepatitis in both autologous and allogeneic patients represented 3% of all patients in our study.

In a recent retrospective study by Torres et al. at MD Anderson who included 59 patients (14 autologous and 13 allogeneic HSCT), all of his patients were HCV-infected recipients, and HCV reactivation was seen in 11% of patients infected with genotype 1; median time to HCV reactivation was 41 days after transplant [12].

In our study, viral hepatitis reactivation had a statistically insignificant effect on liver injury neither during nor post-transplant in both allogeneic and autologous patients. For patients with HBV, the usage of lamivudine prophylaxis starting before the conditioning regimen might be responsible for the reduced risk of reactivation as previously reported by Lau et al. [13]. Similarly, in a study by Firpi and Nelson, they found that prophylactic lamivudine dramatically reduced the chance of reactivation (from 24–53% to 0–5%) and also led to improvement in survival-free from hepatitis [14].

Our results showed that 46.6% of acute hepatic GVHD patients were HCV positive. While 16.6% of patients with chronic hepatic GVHD were HCV+ve and similarly, 16.6% had HBV infection.

Prevalence of CMV infection in our center in allogeneic patients was 57% ( most of them were detected with weekly screening for CMV with no evidence of CMV disease for pre-emptive therapy) with reactivation occurred in about 65% of those who had primary infection, while the prevalence of CMV infection in autologous patients was about 18%. CMV hepatitis reactivation had a statistically insignificant effect on hepatic injury neither during nor post-transplant in both autologous and allogeneic patients. In a retrospective study done by Piukovisc et al., the prevalence of CMV in autologous patients was 24% [15]. While Panagou et al. studied the prevalence of CMV in allogeneic patients, 48% of his patients at least developed 1 episode of CMV viremia during the first 90 days post-transplant [16].

The risk of infection in the post-engraftment period is a function of the dynamics of immune reconstitution. Factors that delay immune reconstitution following HSCT are related to the increased risk of infection. The most important factors influencing the speed of immune reconstitution are the immune status before HSCT and the need for additional immunosuppressive treatment. Likewise, additional chemotherapy after HSCT greatly increases the risk of infection [17]. When we correlated between septicemia and hepatic injury between autologous and allogeneic patients, we found that septicemia had no statistically significant effect on liver injury during transplant. On the other hand, a statistically significant effect of septicemia on liver injury post-transplant was observed. Sepsis was higher in allogeneic patients due to prolonged use of immunosuppressive therapy and chronic GVHD.

## Conclusion

Conditioning regimens, acute, and chronic hepatic GVHD in our study are major causes of hepatotoxicity following allogeneic stem cell transplantation while drug-induced hepatotoxicity, flare of viral hepatitis, and sepsis represent the most common causes of hepatic dysfunction following autologous stem cell transplantation. Determination of liver dysfunction could be determined in many cases with the use of simple non-invasive tests in conjunction with the clinical settings. Effective pre-transplant eradication of viral hepatitis could possibly prevent short and long-term hepatic complications following transplantation.

## Data Availability

The datasets used and/or analyzed during the current study are available from the corresponding author on reasonable request.

## Abbreviations

GVHD: Graft versus host disease
HCT-CI: Hematopoietic cell transplantation comorbidity index
HSCT: Hematopoietic stem cell transplantation
SOS: Sinusoidal obstruction syndrome
